# Evaluating the Effect of the Schroth Method on Sensorimotor Control in Adolescents with Idiopathic Scoliosis: A Controlled Clinical Trial

**DOI:** 10.3390/jfmk11010127

**Published:** 2026-03-21

**Authors:** Alexandros Kastrinis, Nikolaos Strimpakos, George A. Koumantakis, Dionysios Tzatzaliaris, Marianna Oikonomaki, Zacharias Dimitriadis

**Affiliations:** 1 Health Assessment and Quality of Life Research Laboratory, Department of Physiotherapy, Faculty of Health Sciences, University of Thessaly, 35132 Lamia, Greece; alexkastrinis@uth.gr (A.K.); nikstrimp@uth.gr (N.S.); 2 Research Laboratory of Advanced Physiotherapy, Department of Physiotherapy, University of Western Attica, 12243 Athens, Greece; gkoumantakis@uniwa.gr; 3 Scoliosis Spine Laser Center, Moschato, 18345 Attica, Greece

**Keywords:** Schroth, scoliosis, proprioception, joint position sense, repeatability, spine, balance, footplate, Fukuda, sensorimotor control

## Abstract

**Background**: Adolescent idiopathic scoliosis (AIS) is often associated with central nervous system disorders and deficits in sensorimotor function. While the Schroth method is a common clinical intervention, research evidence regarding its effectiveness in enhancing sensorimotor control remains limited. This study aimed to evaluate the impact of the Schroth method on sensorimotor control and quality of life (QoL) in AIS patients. **Methods**: Sixty female participants (mean age 13.4 years) with Cobb angles between 10° and 45° were divided into an intervention group (*n* = 30), receiving Schroth exercises and bracing for 10 weeks, and a control group (*n* = 30), receiving bracing alone. Outcome measures included static and dynamic balance, spine lateral flexion joint position sense (JPS), upper-limb functional proprioception, and the GR-BSSQ Brace questionnaire. **Results**: Statistical analysis using two-way mixed ANOVA revealed significant Group × Time interactions across several parameters. The Schroth group showed significant improvements in static and dynamic balance, with ellipse area reduction (*p* = 0.005) and reduced Fukuda test distance (*p* = 0.007), respectively. Significant enhancements were noted in spine lateral flexion JPS (Bilateral *p* = 0.008) and upper-limb proprioception (Bilateral *p* = 0.000). Furthermore, the intervention group reported a significant improvement in QoL scores compared to the control (*p* = 0.000). **Conclusions**: The findings demonstrate that the Schroth method was associated with enhanced sensorimotor control, supporting its use as a targeted approach to improve functional outcomes in individuals with AIS. These results highlight the clinical value of the method, beyond spinal curve correction.

## 1. Introduction

Adolescent idiopathic scoliosis (AIS) is defined as a structural lateral curvature of the spine with a 10° or greater Cobb angle occurring in otherwise healthy children between the ages of 10 and 18 [1]. AIS is fundamentally a three-dimensional spinal deformity characterized by a lateral curvature in the coronal plane, rotational deformity in the axial plane and sagittal plane alterations affecting normal spinal curvatures [2].

The condition typically manifests during periods of rapid skeletal growth, particularly during the adolescent growth spurt. The deformity can occur in the thoracic, lumbar, or thoracolumbar regions, with thoracic curves being more prevalent [3].

AIS affects approximately 2–4% of children aged 10–16 years, making it the most common form of scoliosis [4]. There is a notable female predominance, particularly among patients whose curves progress and require treatment, with a female-to-male ratio ranging from 1.5:1 for small curves to 8:1 for curves requiring surgery [5].

While the exact etiology remains unknown, AIS is believed to result from complex interactions between genetic, biomechanical, and environmental factors. Current research suggests the involvement of genetic predisposition, hormonal influences during puberty, asymmetric growth patterns and neuromuscular control abnormalities [2]. Untreated progressive curves may lead to cosmetic concerns and psychological impact, respiratory compromise in severe thoracic curves, back pain in adulthood and functional limitations [6].

Patients with AIS consistently demonstrate measurable sensorimotor control deficits, particularly in domains such as static and dynamic balance, lower extremity joint position sense, and cervical spine proprioception [7,8].

In a study conducted by Guyot et al. (2016) [9], the ability of AIS subjects to accurately reposition their heads to a neutral alignment without visual input was evaluated using the cervicocephalic relocation test. The investigators documented the presence of proprioceptive deficiencies in individuals with scoliosis compared to healthy controls [9].

Studies investigating the ability of scoliotic participants to reproduce predetermined angles in the knee and elbow joints found that healthy controls were more accurate in reproducing the target angle [10].

Furthermore, a spatial orientation assessment was used to evaluate the proprioceptive precision of the upper extremities across four distinct cohorts of patients (including AIS patients exhibiting progressive curves and non-progressive spinal asymmetry) [11]. The findings indicated that both the AIS group and the spinal asymmetry cohort exhibited lower accuracy than the control groups.

Dynamic balance deficits are particularly pronounced, with AIS patients showing significantly larger displacement distances and angles of rotation during the Fukuda stepping test (*p* < 0.01 and *p* < 0.0001, respectively) compared to controls, even when static balance tests remain normal [12].

Force plate assessments further demonstrate increased postural instability in AIS, with greater center-of-pressure range, velocity, and sway area in both eyes-open and eyes-closed conditions (all *p* < 0.001) [13]. Collectively, these findings establish that AIS patients possess clinically relevant sensorimotor deficits that alter postural control strategies.

The Schroth method is a Physiotherapeutic Scoliosis-Specific Exercise (PSSE) program aimed at the three-dimensional self-correction of spinal deformity using postural re-education, targeted muscle activation and corrective breathing techniques, taught by certified therapists and as a home program to the patient [14,15]. Developed by Katharina Schroth in the 1920s and later refined by her daughter Christa Lehnert-Schroth, this intensive, pattern-specific rehabilitation approach uses mirror feedback and curve-type classification to guide individualized rehabilitation [14]. The core concept of the method is three-dimensional (frontal, sagittal, transverse) auto-correction with emphasis on rotation and de-rotation through posture and respiratory mechanics [14,15].

The Schroth method integrates sensorimotor retraining, isometric control, postural re-education, and specific breathing techniques to modify trunk geometry and enhance neuromuscular control; therapists individualize these programs according to curve patterns and facilitate home-based practice [15,16].

Current evidence supports the efficacy of Schroth exercises in reducing spinal deformity and improving clinical outcomes. A systematic review and meta-analysis of 14 studies involving 538 patients with adolescent idiopathic scoliosis (AIS), conducted by Chen et al. (2024) [17], suggests that Schroth exercises lead to a significant reduction in both the Cobb angle and the angle of trunk rotation (ATR) compared to conventional physical therapy. Moreover, the intervention was found to improve quality of life and lumbar extensor strength [17].

Consistent findings were reported in a separate systematic review and meta-analysis. This study, which included 114 adolescents with AIS, demonstrated that the Schroth method showed positive effects on the Cobb angle, quality of life, and angle of trunk rotation in the short-term. While the reduction in the Cobb angle reached statistical significance, its clinical relevance requires further consideration [15].

Compelling evidence was provided by a subsequent systematic review and meta-analysis, suggesting that the Schroth method is effective in improving key clinical indicators of idiopathic scoliosis (IS), including the Cobb angle and ATR, while significantly enhancing the quality of life of affected individuals. These results suggest that the Schroth method represents a valuable conservative treatment option for IS. This study included 278 participants, comprising both adolescents and adults ranging from 10 to 38 years of age [18].

The Schroth method integrates postural and sensorimotor exercises to improve a patient’s three-dimensional control over their body, thereby enhancing their ability to maintain corrected spinal alignment. More specifically, in clinical practice, patients are taught to adopt non-scoliotic postures, engage in static and dynamic balance exercises and perform specific corrective exercises. These strategies require the use of proprioceptive feedback from the skin, muscles, tendons and joint capsules. External stimuli, including specialized equipment and tactile feedback provided through corrective movements, offer valuable information, with the addition of visual feedback from mirrors [19].

Although idiopathic scoliosis is theoretically closely associated with deficits in sensorimotor function, and the Schroth method incorporates numerous techniques targeting postural and proprioceptive regulation, the research evidence supporting its effectiveness in enhancing outcomes such as static and dynamic balance, proprioception, and joint position sense remains limited.

The contemporary literature emphasizes that conservative management must evolve beyond purely biomechanical curve correction. A recent study by Campoli et al. (2025) [20] highlights the critical importance of postural and neuromotor approaches that specifically target multisensory integration, sensorimotor recalibration and body schema reorganization. Therefore, the evaluation of clinical interventions should address these broader neurophysiological dimensions to fully capture their therapeutic impact [20].

A pilot study conducted by Akyurek et al. (2022) [21] investigated the effectiveness of the Schroth method on spinal joint repositioning sense. An eight-week Schroth exercise program was administered to adolescents with idiopathic scoliosis (mean age 13.79 ± 1.82 years). Spinal repositioning error was assessed in flexion, extension, and lateral flexion, along with the angle of trunk rotation parameters (ATR), both at baseline and post-intervention. Twenty-nine participants were randomly allocated into two groups. The findings demonstrated that Schroth exercises may improve spinal joint repositioning sense; specifically, significant improvements were observed in thoracic and lumbar joint repositioning error values (*p* < 0.05) [21].

Furthermore, a study by Bayraktar et al. (2018) [22] utilizing a small sample size reported improvements in postural control among patients with idiopathic scoliosis who received conservative treatment consisting of bracing combined with Schroth exercises. Participants in the experimental group wore the brace for 23 h per day and performed individualized Schroth exercises three times per week for 18 sessions. The findings indicated that combining Schroth exercises with bracing may significantly improve specific aspects of postural control, such as the center of gravity (COG) and sway velocity [22].

The combined effects of hippotherapy and Schroth exercises on postural asymmetry and dynamic balance were evaluated in a randomized controlled trial by Abdel-Aziem et al. (2021) [23]. Fifty-two patients with mild idiopathic scoliosis (Cobb angles:10° to 25°) were allocated into two groups. Both groups received Schroth exercises for ten weeks, while the experimental group additionally underwent hippotherapy training. The researchers observed that hippotherapy, when integrated with Schroth exercises, was more effective than Schroth alone in improving postural asymmetry and balance ability in patients with AIS. These improvements suggest that this combination should be considered when developing rehabilitation programs for AIS. Given that scoliosis alters weight distribution, the loading pattern changes depending on curve type, location, and Cobb angle [23].

Two earlier studies compared Schroth exercises with Pilates (Kim & HwangBo, 2016 [24]) and with proprioceptive neuromuscular facilitation (PNF) (Mohamed & Yousef, 2021 [25]) in relation to body weight distribution and static plantar pressure in patients with idiopathic scoliosis. Both studies reported significant improvements in the Schroth groups compared to alternative interventions, suggesting that Schroth exercises are more effective than Pilates or PNF for these specific outcomes [24,25].

Radwan et al. (2022) [26] compared 20 patients with AIS aged 10–16 years with 20 age-matched healthy controls. These researchers utilized the Biodex Balance System to assess the overall stability index (OSI), anterior–posterior stability index (APSI), and medial–lateral stability index (MLSI). Measurements for the clinical group were conducted at baseline, after one month, and after three months of Schroth therapy, while the control group was assessed only once. The findings indicated that Schroth therapy significantly improved OSI, APSI, and MLSI after three months of treatment [26].

Aktan and Erdoganoglu (2021) [27] investigated the effects of the Schroth method following a seven-day intensive program in 45 AIS patients, consisting of 4.5 h of exercise daily. The authors reported positive effects on postural symmetry, trunk muscle endurance, dynamic balance, perceptions of aesthetic deformity, and health-related quality of life [27].

Notably, to date, no studies have examined the effects of the Schroth on the proprioception of the upper or lower limbs.

While the effectiveness of the Schroth method in reducing the Cobb angle and the angle of trunk rotation (ATR) has been well-documented, there is a limited body of literature focusing on its effectiveness regarding sensorimotor control. Therefore, the primary aim of the present study is to investigate the impact of a 10-week Schroth intervention on sensorimotor control, specifically addressing upper-limb proprioception, spinal joint position sense, and static and dynamic balance in adolescents with idiopathic scoliosis. A secondary aim is to examine the effect of this method on the quality of life of these individuals.

## 2. Materials and Methods

### 2.1. Study Design and Sample

A total of sixty participants diagnosed with AIS enrolled in this single-blind, controlled clinical trial. The volunteers were recruited from the patient pool of Scoliosis Spine Laser Center, Moschato, Greece, and were divided into two groups of thirty individuals based on physician prescription and the family-centered decision-making model [28]. The sample size calculation was performed using G*Power 3.1.9.4 [29]. Based on the intended statistical analysis (ANOVA 2 × 2) to identify a large effect size (f = 0.4), with a significance level of α = 0.05 and a statistical power of 80%, the required sample was determined to be at least 52 individuals (26 per group). Assuming a 10% attrition rate, the proposed sample size was set at 58 individuals. Therefore, 30 individuals per group were included in the study.

The experimental group received bracing treatment with a Schroth-compatible brace and followed a Schroth-based program for 10 weeks, whereas the control group received bracing treatment alone. Participants were included if they met the following criteria: (a) female sex, (b) age between 10 and 16 years, (c) Risser sign between 0 and 4, (d) primary curve with a Cobb angle between 10° and 45°, (e) primary right thoracic curve according to Schroth classification [30], (f) bracing treatment performed with a Scoliosis Brace by SLC and (g) fluent in the Greek language. Volunteers were excluded if they suffered from (a) musculoskeletal or neurological diseases affecting motor control or (b) balance, (c) cognitive problems, or (e) mental illnesses, or they (f) had previous treatment with a brace or any form of exercise for their condition or (g) had any vestibular pathologies unrelated to scoliosis.

All participants and their legal guardians provided written informed consent prior to enrollment in the study. The study was conducted in accordance with the guidelines of the Declaration of Helsinki and was approved by the Ethics Committee of the Physiotherapy Department, University of Thessaly, Lamia, Greece (Protocol No. 41/7 April 2023). Furthermore, the study was registered with the ISRCTN clinical study registry (ISRCTN24878103, Registration date: 26 September 2024).

### 2.2. Equipment, Materials and Outcome Measures

Full-spine radiographs (frontal and sagittal planes) were obtained for all the participants. Based on these radiographs, the initial Cobb angle of the primary curves was calculated, and the Risser sign, which relates to the level of skeletal maturity, was recorded. The Cobb angle calculation method has been reported as reliable across various evaluators, including experienced physicians (ICC = 0.94) and rheumatologists (ICC = 0.91–0.95) [31]. The Risser sign has been reported to correlate significantly with both chronological age (r = 0.791 for girls and r = 0.787 for boys with AIS) and the TW3 skeletal maturation test (r = 0.718 for girls and r = 0.785 for boys with AIS) [32]. The primary curve type was determined according to the Schroth methodology, which incorporates clinical observation, special flexibility tests of the spine, and the identification of a radiological index called “Transitional Point”, to categorize the specific curve pattern [30].

Quality of life (QOL) was evaluated using the GR-BSSQ Brace questionnaire. This questionnaire consists of eight items, and its original German version (Botens-Helmus et al., 2006) demonstrated excellent internal consistency (Cronbach’s α = 0.97) and adequate test–retest reliability (ICC = 0.88) [33]. The questionnaire has been cross-culturally adapted and validated for the Greek population, demonstrating good internal consistency with (Cronbach’s α = 0.87) and excellent test–retest reliability with an ICC of 0.94 (95% CI 0.89–0.97) [34].

Dynamic balance was assessed using the Fukuda stepping test. This test has shown sufficient reliability in healthy populations (ICC = 0.66 for the angle of rotation from the starting position and ICC = 0.69 for the distance from the starting position) using a 50-step protocol [35]. Furthermore, the Fukuda test has been evaluated for reliability in patients with AIS, demonstrating good test–retest reliability, particularly regarding the distance from the starting point. Specifically, the mean values of the second and third trials for absolute error measurement showed better reliability, with an ICC of 0.85 (95% CI: 0.56–0.85), a standard error of measurement (SEM) of 15.03, and a smallest detectable difference (SDD) of 41.64 [36].

Static balance was evaluated based on sway area and sway velocity parameters using a force footplate (model EPS + R, Loran Engineering, Bologna, Italy) and the associated software. Barozzi et al. (2014) evaluated the test–retest reliability of sway area and sway velocity in healthy schoolchildren aged 6 to 14 years, reporting moderate reliability (ICC = 0.57 and 0.61 for sway area with eyes open and eyes closed, respectively) and high reliability (ICC = 0.75 and 0.76 for sway velocity with eyes open and eyes closed, respectively) [37]. Kastrinis et al. (2024) [36] evaluated the test–retest reliability of these fundamental static balance components in patients with AIS. Their analysis demonstrated that the mean values of the first two trials exhibited good reliability for sway velocity (ICC (95% CI) = 0.74 (0.23–0.91), SEM = 6.37, SDD = 17.66) and for the center of gravity (COG) ellipse area (ICC (95% CI) = 0.74 (0.27–0.91), SEM = 142.46, SDD = 394.43) [36].

Spinal joint position sense (JPS) assessment using a single digital inclinometer has been previously established in the literature [38]. That study involved two investigators and two groups of participants, comprising 30 healthy individuals and 30 patients with low back pain. The findings suggested good to excellent reliability for both neutral and target lumbar positioning, with intra-rater and inter-rater ICC values ranging from 0.75 to 0.93. Participants with low back pain exhibited significantly greater proprioceptive errors compared to healthy controls (*p* < 0.001). In the present study, a spinal lateral flexion JPS test was conducted using a digital inclinometer (Digital Level Box, eSync, Hong Kong, China). The inclinometer was fixed to a predetermined anatomic location using double-sided adhesive tape (DS1925, HPX, Temse, Belgium). The spinal lateral flexion JPS test demonstrated good to excellent test–retest reliability for both the constant and absolute errors on the left and right sides. Specifically, ICC values ranged from 0.83 to 0.95, with SEM values between 0.93 and 1.53 and SDD values ranging from 2.59 to 4.24. Statistical calculations indicated that 10 trials are required for this test to achieve a SEM value equal to 20% of the grand mean [36].

Based on the description provided by Keessen et al. (1992) [11], a panel-shaped instrument was constructed to evaluate upper-limb proprioception. The instrument (30 × 40 cm) comprised two bonded transparent acrylic glass panels with thicknesses of 5 cm and 3 cm for the inferior and superior panels, respectively. Prior to assembly, eight apertures (8 mm in diameter) were drilled into the inferior panel to facilitate index finger insertion and stabilization under the panel. This configuration prevented the participant’s contralateral index finger from physically contacting the target, which represented the exact position of the index finger located beneath the panel. The apparatus was mounted on a tripod (Nedis TPOD2200GY, ’s-Hertogenbosch, The Netherlands) to align the panel height with the participant’s shoulder level. The apertures were symmetrically positioned from the center of the panel at a radial distance of 10 cm. A ninth aperture was positioned at the center of the instrument for familiarization purposes. Apertures 1, 4, and 6 were proximal to the participant’s torso, while apertures 3, 5, and 8 were distal. Apertures 2 and 7 were located at an intermediate distance. A modified sewing thimble (Iris Sewing, Taizhou, China) was utilized to cover the moving index finger; a 2 mm aperture was drilled into the tip of the thimble, allowing the assessor to mark the final resting position of the index finger with a pen. Although a similar test has been previously used to investigate upper-limb proprioceptive accuracy in patients with AIS [11], a subsequent study [36] established the measurement properties of the test. According to that study, the upper extremity functional proprioception test demonstrated excellent test–retest reliability for the total test score (ICC (95% CI) = 0.90 (0.71–0.96), SEM = 0.47, SDD = 1.3). For the left and right upper extremities, test–retest reliability was found to be good with (ICC (95% CI) = 0.86 (0.40–0.91), SEM= 0.47, SDD= 1.3) and (ICC (95%CI) = 0.87 (0.63–0.95), SEM = 0.67, SDD = 1.87) values, respectively. The mean values of the left and right sides, as well as the combined average (R + L), were calculated as the aggregate of the separate measurements from apertures 1 through 8.

All distance measurements were obtained using a (Seca GmbH & Co. KG, Hamburg, Germany) tape measure, and for all eyes-closed tests, a standardized adjustable blindfold (Yunmoxiao, Shenzhen, China) was used to eliminate visual input.

To ensure treatment standardization, participants were fitted with the same type of orthosis: a three-dimensional, Schroth-compatible, asymmetrical brace manufactured by the Scoliosis SLC Center (Moschato, Greece) [39,40]. The fitting was performed by certified technicians, and both patients and their parents received standardized instructions. Trunk geometry was acquired using a portable 3D laser scanner (Fast Scan Cobra, Polhemus; Aranz Scanning Ltd., Christchurch, New Zealand). Brace design and contouring were conducted by the same orthotist, using CAD/CAM technology according to an established protocol [41]. The brace model was generated using specialized software (Rodin4D NEO Neo 2018 (10.2), Rodin4D Ltd., Mérignac, France) and tailored to patient-specific anthropometric data and the physician’s prescription. The orthoses were subsequently manufactured using an automated milling system (FR4D-S, Rodin4D Ltd., Mérignac, France).

### 2.3. Procedure

**Figure 1:**
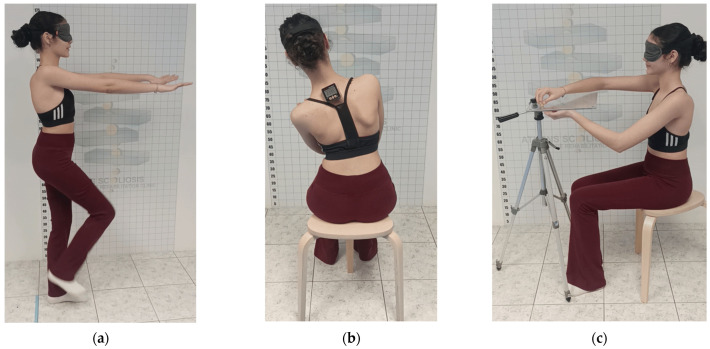
Sensorimotor control tests. (**a**) Fukuda test, (**b**) spinal lateral flexion joint position sense test and (**c**) upper-limb functional proprioception test.

The participants and their parents provided written informed consent prior to enrollment in the study. Demographic data were acquired, scoliosis curve patterns were classified according to the Schroth methodology, and participants were allocated to the intervention or control group based on physician prescription and participant wishes. A physical therapist, experienced in scoliosis treatment, was responsible for the baseline and follow-up assessments after the 10-week period. The therapist was blinded to the group allocation of each participant. At baseline, participants completed the GR-BSSQ questionnaire. The BSSQ Brace evaluates brace-induced stress and consists of eight Likert-scale items. Scoring ranged from 0 (worst) to 3 (best). Score interpretation was categorized as follows: 0–8 indicates high stress, 9–16 moderate stress, and 17–24 low stress [34]. Four sensorimotor control tests were utilized, following the protocol described by Kastrinis et al. (2024) (Figure 1) [36]. All tests were performed with eyes closed; however, participants completed an eyes-open trial prior to the assessment for familiarization purposes. A 15 min rest interval was provided before the implementation of each test.

Static balance was assessed first. To determine the sway velocity and the center of gravity (CoG) ellipse area, the participant stood barefoot for 30 s on the force plate with their heels 10 cm apart. Participants were instructed to gaze at a fixed point located one meter (1 m) in front of them and maintain their head position during the eyes-closed trials. Two eyes-closed trials were conducted [36].

Subsequently, participants performed the Fukuda test. The participant’s initial standing posture was marked, after which they were instructed to flex both shoulders to a 90° angle and maintain this position. With their eyes closed, the participant was directed to perform steps in place with a hip flexion angle of approximately 45°. Following 50 steps, the distance traveled from the initial position was measured in centimeters, and the angle of rotation in relation to the starting position was recorded. This procedure was conducted three times; however, based on established reliability data [36], only the measures from the second and third trials were included in the analysis.

The subsequent assessment was the spinal lateral flexion JPS test. From a seated position, the participant was directed to maintain the pelvis in a neutral alignment and position their arms across their chest with the palms oriented towards the shoulders. The participant was guided at a slow and consistent pace to a 20° angle of lateral flexion with eyes open. This position was held for five seconds to facilitate proprioceptive encoding, after which the participant returned to the initial posture. Following this phase, the participant performed active reproduction of the target angle. The evaluation was repeated ten times for each side. During each trial, the deviation (absolute error) between the repositioned angle of lateral flexion and the target angle was recorded. For this purpose, a digital inclinometer was positioned over the C7 vertebra throughout the assessment [36].

The final assessment comprised the upper extremity functional proprioception test. This test was conducted with the participant in a seated position with their eyes closed. Following a randomized sequence, the index finger of one hand was placed into one of the apertures from beneath the transparent apparatus. The participant was then instructed to locate, utilizing the index finger of the contralateral hand from above the apparatus, the precise position of the index finger positioned below. The examiner quantified the linear distance between the participant’s index fingers during each trial. This procedure was repeated three times for each of the eight apertures, and identical measurements were obtained for both upper extremities.

All assessments were repeated by the same blinded assessor following the 10-week intervention period.

**Figure 2:**
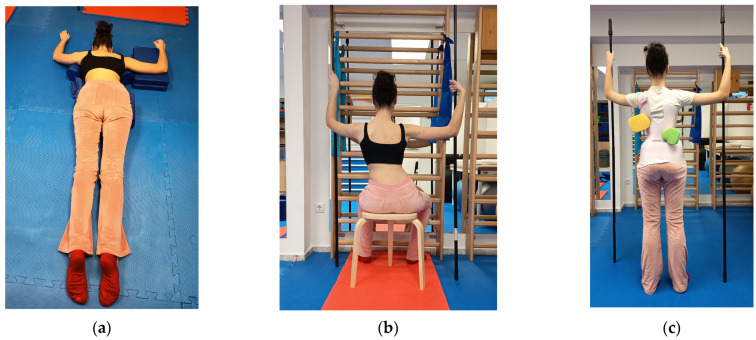
Schroth exercises for primary thoracic curves. (**a**) Shoulder counter traction in the prone position, (**b**) sitting on a chair and (**c**) between two poles standing while in the brace; sponges are used to stimulate breathing zones.

In addition to full-time bracing, the intervention group followed a Schroth-based protocol. The program was conducted twice weekly under the supervision of an experienced Schroth therapist for one hour, supplemented by a home exercise program performed five times per week for 30 min. The Schroth program was designed to address the primary curve and any additional compensatory patterns [30]. The supervised sessions included Schroth-specific mobilizations, pelvic control exercises, postural awareness exercises, three-dimensional (3D) breathing, and standard over-corrective Schroth exercises (Figure 2) performed in supine, sitting, kneeling, and standing positions. Specific maneuvers included shoulder counter traction (supine, prone, and side-lying), “sitting on a chair,” “chest twister,” “sail” (kneeling and standing), and “between two poles” (standing). In-brace exercises were also incorporated [42]. The home exercise program entailed simplified versions of these exercises to minimize technical errors. To facilitate compliance, the therapist filmed the exercises on the participants’ smartphones, providing digital files for reference. Additionally, the home program focused on maintaining non-scoliotic postures while out of the brace, along with education on adopting these postures during activities of daily living (ADLs) [43].

### 2.4. Statistical Analysis

Data analysis was performed using IBM SPSS v.25. The normality assumption was assessed using the Shapiro–Wilk test and visual inspection of Q-Q plots [44]. Descriptive statistics are presented as means and standard deviations (SDs).

Baseline equivalence between the two groups for all demographic, clinical, and primary sensorimotor variables was evaluated using independent samples *t*-tests (or Mann–Whitney U tests for non-parametric data). A paired-samples *t*-test was used for the analysis of within-group changes between baseline and post-treatment at 10 weeks for each group. A two-way mixed ANOVA was conducted to determine the Group × Time interaction effect for each one of the dependent variables. The level of significance for all tests was set at *p* < 0.05.

## 3. Results

### 3.1. Baseline Characteristics and Compliance

**Figure 3:**
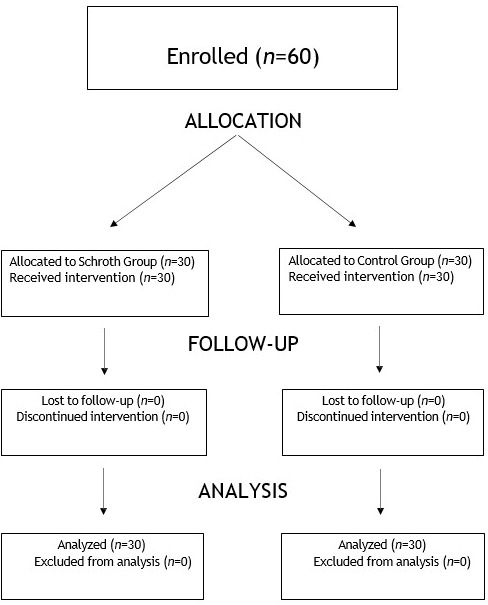
TREND participant flow diagram.

Baseline demographics and clinical characteristics for the sixty participants are summarized in Table 1. The flow of participants through each stage of the trial (enrollment, allocation, follow-up, and analysis) is detailed in Figure 3. The two cohorts exhibited statistical similarity across all initial parameters, with no significant differences observed in age, height or weight. Furthermore, the groups were comparable regarding skeletal maturity as indicated by Risser stage (*p* = 0.54) and the magnitude of the Cobb angle of the primary curve (*p* = 0.21).

Statistical analysis confirmed that the intervention and control groups demonstrated baseline equivalence across the majority of the sensorimotor outcome measures (*p* > 0.05). Isolated baseline differences (e.g., in left upper-limb proprioception and Fukuda angle) were inherently accounted for by the two-way mixed ANOVA design.

Self-reported compliance rates were recorded for both groups over the intervention period. In the intervention group, the mean compliance for brace wear was 73.8% (±16.6) and compliance for the prescribed Schroth exercise program 78.3% (±18). In the control group, the mean bracing compliance was 73.2% (±25.3). An independent samples analysis revealed no statistically significant difference in brace wear compliance between the two groups (*p* > 0.05).

### 3.2. Quality of Life and Brace-Related Stress

For the GR-BSSQ Brace, within-group comparisons showed a significant improvement in brace-related stress levels (*p* = 0.000), while no changes were observed in the control group (Table 2).

In Table 3, a clear migration from high/medium stress towards low/medium stress can be observed in the Schroth group, while relative stability can be observed in the control group, with most participants remaining at the medium stress level throughout the study.

**Figure A1:**
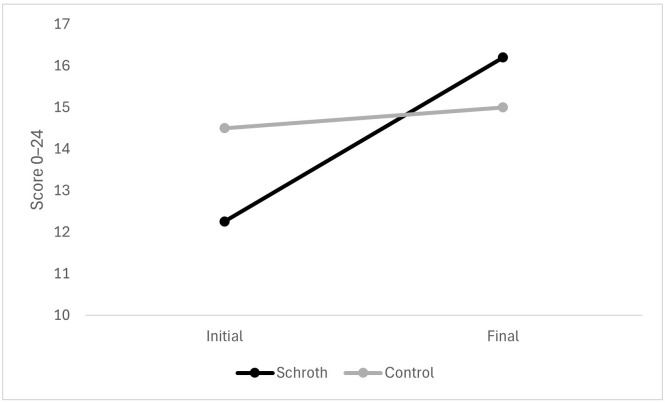
Changes in GR-BSSQ Brace scores over time.

A two-way mixed ANOVA revealed a significant (*p* < 0.001) Group × Time interaction for the brace-induced stress levels and a large effect size (Partial Eta^2^ = 0.276), suggesting the change over time was significantly different between the two groups (Table 4). This Group × Time interaction is illustrated in Figure A1, clearly showing a different pattern of scoring between the two groups over the study period.

### 3.3. Static and Dynamic Balance Outcomes

For the static balance test (sway velocity), a non-significant improvement was observed (*p* = 0.065) in the Schroth group, while no changes occurred in the control group (*p* = 0.893). In contrast, there was a statistically significant change in the Schroth group for the ellipse area component (*p* = 0.000), but no changes were observed in the control group (*p* = 0.388) (Table 5).

**Figure A2:**
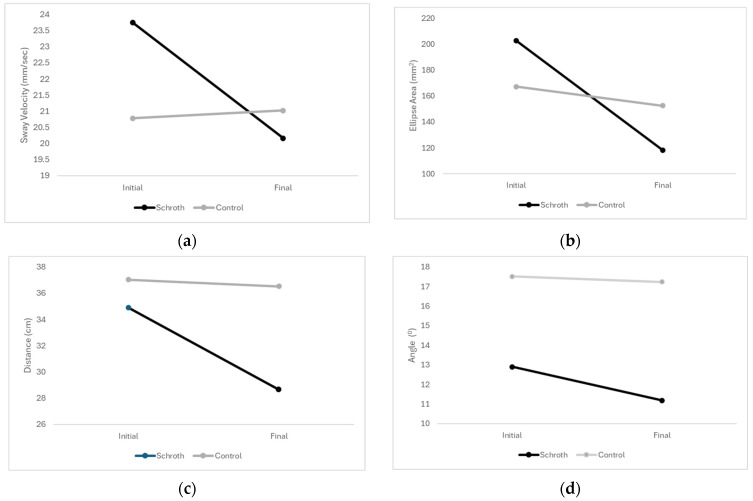
Visual illustration of Group × Time interactions for the static and dynamic balance. (**a**) Changes in sway velocity over time, (**b**) changes in ellipse area over time, (**c**) changes in Fukuda distance over time and (**d**) changes in Fukuda angle over time.

The ANOVA analysis (Table 6) revealed no significant Group × Time interaction for the sway velocity component of the static balance test (*p* = 0.148). The ellipse area component exhibited a significant Group × Time interaction (*p* = 0.005) and a moderate effect size (Partial Eta^2^ = 0.126), indicating improved stability for the Schroth group. These results are further illustrated in Figure A2a,b, highlighting the different trajectories between groups over time.

For the two components of the Fukuda test, within-group comparisons (Table 7) demonstrated a statistically significant change in the distance component in the Schroth group (*p* < 0.001). In contrast, the angle component (*p* = 0.428) in the intervention group and both components in the control group demonstrated no statistically significant changes.

The Group × Time effects reported in Table 8 show a significant interaction for the distance component of the Fukuda test (*p* = 0.007) with a moderate effect size (Partial Eta^2^ = 0.118). The interaction for the angle component was not statistically significant (*p* = 0.567). Figure A2c,d visualize the changes over time.

### 3.4. Proprioception Outcomes

For the spinal lateral flexion JPS test, the Schroth group exhibited significant improvements, as shown in Table 9. These improvements were observed for both the right and left sides (*p* = 0.001 and *p* = 0.04, respectively) and their combined means (*p* < 0.001). The control group did not demonstrate any significant changes (right side *p* = 0.625, left side *p* = 0.848 and combined means *p* = 0.651).

**Figure A3:**
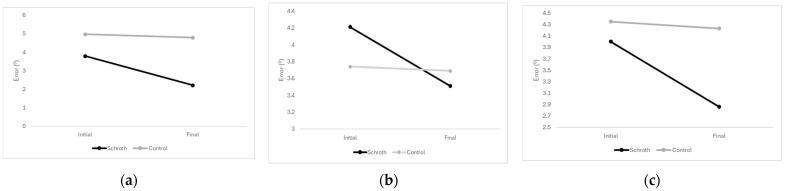
Visual illustration of Group × Time interactions for the spinal lateral flexion joint position sense test. (**a**) Changes in right spinal lateral flexion joint position sense test over time, (**b**) changes in left spinal lateral flexion joint position sense test over time and (**c**) changes in combined spinal lateral flexion joint position sense test over time.

Table 10 of the ANOVA analyses reveals a significant (*p* = 0.014) Group × Time interaction for the right-side JPS error and a moderate effect size (Partial Eta^2^ = 0.100). The combined Group × Time interaction is also significant (*p* = 0.008) with a moderate effect size (Partial Eta^2^ = 0.117). In contrast, the left-side JPS error Group × Time interaction did not show significant changes (0.097). The data for this parameter are illustrated in Figure A3b. Figure A3a,c visually demonstrate the different trajectories over time between the two groups and the magnitude of change that occurred in the Schroth group.

For the upper-limb functional proprioception test, within-group comparisons (Table 11) reveal significant improvements for the Schroth group in both the right upper limb (*p* < 0.001) and the left upper limb (*p* = 0.001) as well as for their combined means (*p* < 0.001). On the contrary, the control group demonstrates no reduction in the error. The data presented in this table represent the means derived from apertures 1 to 8 for each upper limb as they appear in Table A1 and Table A3.

**Figure A4:**
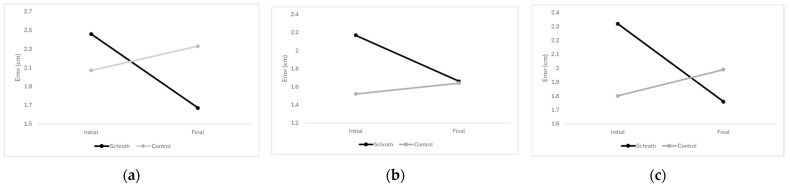
Visual illustration of Group × Time interactions for the upper-limb functional proprioception test. (**a**) Changes in right functional proprioception test over time, (**b**) changes in left functional proprioception test over time and (**c**) changes in combined functional proprioception test over time.

Table 12 demonstrates significant Group × Time interactions for the Schroth group. More precisely, both the right and left sides reduced the combined error (*p* < 0.001) with large effect sizes (Partial Eta^2^ = 0.260, Partial Eta^2^ = 0.198 and Partial Eta^2^ = 0.316, respectively). The data presented in this table represents the means derived from apertures 1 to 8 for each upper limb as they appear in Table A2 and Table A4. Figure A4a–c demonstrate the trajectories of the lines for both groups over time, clearly depicting the error reduction for the Schroth group.

## 4. Discussion

This controlled clinical trial demonstrates that a 10-week Schroth program, when added to bracing treatment, significantly enhances sensorimotor control in adolescents with AIS. Additionally, brace-related stress was significantly reduced in the experimental group. The two groups at study entry were comparable (Table 1) in terms of clinical and demographic characteristics.

The comparable self-reported brace compliance rates between the two groups can be attributed to several clinical and methodological factors. First, both groups received the same standardized baseline approach from their orthotist. This included education and information regarding the critical importance of brace wear. Second, because all participants were at the same phase of their treatment, they likely exhibited comparable adherence [45]. The qualitative literature suggests that brace compliance typically follows a phasic pattern, being relatively stable during the initial and mid-term period following prescription, before the gradual brace fatigue [46]. Finally, the awareness of being monitored due to the clinical trial participation may have influenced the participants to maintain or report similar adherence levels [47]. This similar behavior in terms of brace compliance supports the notion that any observed differences in clinical outcomes can be attributed to the exercise intervention rather than discrepancies in brace usage. Regarding the Schroth exercise program, a strong level of adherence was demonstrated by the participants of the intervention group. This provides a solid basis for the significant sensorimotor and psychosocial improvements observed in this group.

The static balance test was the first assessment performed for both groups. Our findings reveal that a significant change occurred regarding the reduction in the ellipse area over time in the Schroth group, indicating that the program potentially improves the participants’ ability to maintain a more stable center of gravity (COG). However, considering that the previous literature [36,37] reports only moderate test–retest reliability for sway area parameters in adolescent populations, the clinical relevance of these specific static balance improvements should be interpreted cautiously. The sway velocity parameter showed a trend towards improvement, but this change was not significant enough. No notable changes were observed in the control group. Previous findings [48] suggest that these two components are not always linked and reflect distinct strategies of postural control. Several studies also suggest that an intervention may reduce area measures without affecting velocity [49] and that parameters related to the COG area are usually more responsive to interventions compared to velocity, which might remain stable [50]. Studies in healthy children, adolescent and young adult populations suggest that different kinds of training can influence different balance parameters. More precisely, static tasks focused on proprioceptive awareness improve sway area parameters [51,52], while high-difficulty reaction tasks may improve sway velocity parameters [52,53]. The Schroth method is a more statically oriented treatment, which would explain this result. A previous study involved 20 volunteers divided into two groups: 10 patients with AIS and 10 healthy adolescents. A force platform was used to assess postural control under different conditions. Measurements were taken with eyes open and closed on firm and soft surfaces. The experimental group was treated with full-time bracing and three supervised Schroth sessions per week for 6 weeks. The AIS group also performed a Schroth home exercise program. Data was collected at baseline, at 6 weeks and at 18 weeks. Significant sway velocity differences were identified at baseline between the AIS group and their healthy peers. Post-treatment measures revealed a normalization of sway velocity with no significant differences between the two groups (*p* > 0.05). The study did not report sway area as an outcome measure [22]. A case series study involving 23 patients with AIS treated with Schroth and bracing assessed postural parameters with a force plate. The duration of the intervention was three months. Significant sway velocity and sway area improvements were noted (*p* < 0.001) [50]. These findings also suggest the effectiveness of Schroth on postural control. Based on the data from this more recent study [54], it might be suggested that a longer treatment time is necessary for significant changes in sway velocity to be established.

In terms of dynamic stability, a selective improvement pattern appeared in the Fukuda test. The Schroth group demonstrated a significant reduction in displacement from the starting point, while the angle aspect of the test remained unchanged. The control group did not demonstrate any significant changes in either parameter. The ANOVA analysis confirmed a significant Group × Time interaction. These results suggest that the Schroth intervention, when added to bracing treatment, successfully enhanced certain components of dynamic postural stability under eyes-closed conditions. The Schroth method promotes three-dimensional postural correction through sustained positions, which facilitates proprioceptive awareness [55]. Schroth exercises, according to the curve pattern, use targeted asymmetrical muscle activations to promote spinal alignment [56]. We hypothesize that this improved trunk alignment, coupled with enhanced proprioceptive awareness, may have resulted in improved center of mass control during the Fukuda test, leading to a reduction in forward displacement. Regarding the angle component of the Fukuda test, studies suggest that there is substantial intra-individual and inter-individual variability as well as low to moderate test–retest reliability, which limits the sensitivity of the test in short-term treatment [36,57,58]. In addition, stepping tasks under visual deprivation increase reliance on the vestibular system and automatic locomotor control mechanisms [57], and patients with scoliosis often exhibit asymmetrical vestibular processing and alterations in vestibular function [59]. The Schroth method involves spinal de-rotation techniques and angular rotational breathing, but again within the concept of maintaining a stable corrective posture. Limited dynamic rotational tasks are executed during the exercises, and those in the standing position come at a later stage of the program [60]. Recalibrating vestibular mechanisms might require rotation-specific dynamic tasks [61] because they might not be affected by simpler balance and proprioception tasks [62]. The Fukuda test has been used in a previous study as an outcome measure. That study used the SEAS approach as an intervention. Their results demonstrate that patients with AIS treated for a year with the SEAS approach exhibited results statistically similar to the healthy control group, while untreated patients with AIS did not show similar positive results [63].

Assessment of the joint position sense test in spinal lateral flexion indicates that the Schroth group achieved significant improvements in proprioceptive accuracy, especially on the right side and in the combined JPS error. The left-side error also demonstrated a within-group improvement, but the Group × Time interaction indicates that this error improvement cannot be attributed to the intervention. The right-side and combined Group × Time interaction was significant with moderate effect sizes. Patients with AIS demonstrate different paraspinal activity with the convex side being more active, as has been documented with electromyography [64,65]. This pattern seems to be an adaptation to the development of the curve [64,66]. We hypothesize that these asymmetrical, side-specific Schroth exercises may have contributed to the superior results observed on the right side of the Schroth group. The control group did not exhibit any significant changes. Since the concave side has a larger range of motion than the convex [67], it is also theoretically possible that JPS accuracy can be affected accordingly [68]. Nevertheless, given that the Group × Time interaction with the left-side JPS did not reach statistical significance, these mechanistic explanations remain speculative and should be interpreted with caution. Overall, the results support the idea that a Schroth-based program can improve spinal proprioception, which is critical for postural control and movement accuracy. In alignment with contemporary neuromotor paradigms, these enhancements in JPS and spatial awareness likely reflect broader sensorimotor recalibration and body schema reorganization mechanisms, extending beyond strictly localized or biomechanical muscular adaptations [20]. Another study reports positive results in spinal proprioception following a Schroth-based program. Two groups of patients with AIS were recruited: 15 received Schroth treatment and 14 were placed on a waiting list and served as controls. Spinal proprioception was assessed in thoracic and lumbar areas, and the repositioning error for flexion, extension and lateral flexion was measured. The authors report significant within-group and between-group results for all directions with a decrease in repositioning error following an 8-week intervention program [21]. Their conclusion aligns with the findings of this study.

In this study, it is demonstrated that a 10-week Schroth intervention leads to clinically significant improvements in upper-limb proprioception in patients with AIS on both the right and left sides as well as bilaterally. Group × Time significant interactions with large effect sizes provide evidence that these improvements can be attributed to the Schroth intervention. At the same time, no significant improvements were noted in the control group. In AIS rib cage deformity inherently alters scapular kinematics. Specifically, the convex-side shoulder often presents with forward posturing, scapular winging, and anterior tilt, whereas the concave-side shoulder typically remains retracted [36,69]. Consequently, patients with AIS frequently exhibit reduced shoulder functionality compared to healthy peers [69]. Schroth treatment aims to improve spinal curvature, trunk rotation and trunk symmetry utilizing core elements such as three-dimensional autocorrection, three-dimensional breathing and motor control training [15,18,70]. Research demonstrates that Schroth can improve trunk symmetry, spinal alignment and the angle of trunk rotation. This improvement facilitates better scapular and shoulder kinematics [14,15,18]. The shoulder girdle is actively involved during Schroth exercises when a thoracic curve exists using shoulder counter-traction [71]. This entails actively leveling the shoulders and using the shoulder on the concave side as a lever to pull the deviated spine towards the midline and open the collapsed area [60], while on the convex side, the shoulder and scapula are used as fixed points to push the rib hump forward while maintaining the scapular abduction position [72]. The clinical benefits of Schroth and active shoulder girdle involvement seem to improve upper-limb proprioception. While the magnitude of these improvements supports the hypothesis that curve-specific rehabilitation positively influences global sensorimotor integration, a methodological parameter should be acknowledged. Because the control group received bracing only, the present study design cannot definitively isolate whether these specific sensorimotor benefits are exclusive to the Schroth method or if they could partially arise from any structured therapeutic exercise. Therefore, the external validity of the results to other physiotherapy treatment approaches is something that cannot be supported by the current study, and it necessitates further investigation with different treatment protocols.

While bracing is an effective treatment for controlling AIS progression [73,74], it may have a negative psychological impact on patients with AIS. Adolescents under bracing treatment often feel that this might affect their relationships and acceptance by their peers, as well as their ability to participate in sports; in some cases, this can cause isolation and depression [75]. Research suggests that brace-induced stress may affect treatment adherence, leading to a higher risk of scoliosis progression [76,77]. This study’s secondary objective was to evaluate the level of brace-induced stress. The findings demonstrate a significant reduction in stress for the Schroth group. The control group did not demonstrate any significant changes during this 10-week period. The study findings suggest that the Schroth method, apart from its physical benefits, may also provide a support mechanism during treatment that enhances quality of life. In the present study, the intervention group naturally experienced significantly more direct interaction with a specialized physical therapist. Previous studies suggest that increased interaction time fosters a strong therapeutic alliance, which might be a predictor of improved clinical outcomes, enhanced quality of life and better treatment adherence [78,79]. In this context, the supportive therapeutic environment is an important component of the intervention that likely established positive expectations and motivation, contributing to the significant reductions in brace-related stress. It is essential to emphasize, though, the clinical value of the Schroth method’s psychosocial components, particularly how it transforms the patient’s relationship with the brace. Patients learn that the brace is not merely a passive, restrictive device. Instead, they are taught that they can use the brace as a dynamic tool during in-brace exercises, in-brace corrective movements and 3D breathing [42]. This helps the adolescents realize that the brace does not have to limit their physical capabilities. These findings align with those of previous studies suggesting that the Schroth method improves self-image, mental health and health-related QOL in patients with AIS [15,56,80].

From a clinical perspective, the present study demonstrates that these meaningful sensorimotor improvements can be achieved through a highly accessible, clinically applicable exercise-based intervention. The efficacy of this approach is rooted in the strong clinical reasoning of the Schroth method, which emphasizes individualized, active 3D auto-correction and conscious neuromotor control [14,15,19]. Unlike therapeutic approaches that rely heavily on advanced, expensive, or instrument-dependent technologies, this methodology utilizes standard, readily available clinical equipment, highlighting its practical value for the everyday conservative management of the AIS.

### 4.1. Clinical Implications

Radiological monitoring is a very important indicator of treatment success [81], as are measurements of the angle of trunk rotation (ATR) [82]. Studies suggest that in-brace X-rays, for patients undergoing bracing treatment, and the first out-of-brace X-ray may also serve as predictive tools for treatment [43]. The findings of this study suggest that sensorimotor control indexes can also serve as additional quantifiable markers for treatment success. A similar suggestion was made in an RCT study that measured spinal joint repositioning error after an 8-week Schroth-based program [21]. The data imply that the Schroth method can achieve a neuromuscular recalibration and reorganization of the body schema, allowing patients with AIS to better control their posture.

Sensorimotor control deficits are directly correlated with functional limitations in adolescents with idiopathic scoliosis. Studies reveal that patients with AIS exhibit gait asymmetries regarding specific biomechanical markers on the transverse planes, with primary right thoracic curves maintaining gait through compensatory mechanisms [83,84]. Alteration in postural control and balance is characterized by greater fluctuations in the center of pressure and tending to depend disproportionately on ankle proprioception during specific tasks [85,86]. Thus, improving sensorimotor control in patients with AIS is directly connected to functionality.

According to Panjabi (1992) [87], three subsystems interact to achieve spinal stability. The passive subsystem (vertebrae, discs, ligaments, capsules), the active subsystem (muscles, tendons) and the neural control subsystem (proprioceptive sensors, central processing, motor output) [87]. The literature provides evidence that Schroth may influence the passive system by reducing the Cobb angle and ATR [15,17,18,56]. Other studies suggest that the Schroth method may influence the active subsystem by selectively activating muscle groups [64,65]. This study reports improvement in sensorimotor control, which may represent a neuromuscular mechanism contributing to enhanced active spinal stability. Although the current study does not permit establishing a cause–effect relationship between sensorimotor changes and spinal alignment improvements, the findings support the hypothesis that Schroth-based exercises may influence the neural control subsystem.

### 4.2. Limitations

While this study provides new evidence regarding the effect of Schroth on sensorimotor control, there are limitations that need to be acknowledged. A limitation of the present study is the non-randomized design, as group allocation was based on physician prescription and family preferences. While this approach reflects real-world clinical practice, it may introduce a degree of selection bias. For instance, families actively opting for the Schroth method might exhibit higher baseline motivation, which could positively influence subjective measures like the GR-BSSQ Brace questionnaire. Although all participants met the same strict inclusion criteria and demonstrated baseline comparability, the findings—particularly regarding subjective outcomes—should be interpreted with this design characteristic in mind. Importantly, group allocation was not based on a differing clinical prognosis or a greater physical suitability for the therapy, as all participants met the same strict inclusion criteria and possessed an equal theoretical potential for physical improvement. Rather, the assignment strictly reflected the preferences and decisions of the physicians and families. Nevertheless, while the groups were clinically comparable at baseline, the unmeasured psychosocial differences inherent to this non-randomized allocation (such as baseline motivation) cannot be completely controlled.

The sample consisted of female participants only with primary right thoracic curves. This restriction was a deliberate design decision made to ensure maximum sample homogeneity, as this demographic and curve type represent the most prevalent cases in clinical practice. Although the sample reflects the most predominant clinical demographic, as the female-to-male ratio for progressive curves can reach 8:1 [5], this limits the external validity of the study, restricting the generalization of the results to male patients and those with primary lumbar patterns.

Left thoracic curve patterns were excluded since they constitute an atypical clinical presentation that serves as a strong predictor for underlying neural axis abnormalities [88]. While the sample did not include primary lumbar curve patterns, the Schroth classification used in this study identifies subtypes where the primary thoracic deformity is accompanied by a secondary lumbar curve. Schroth exercises are designed to address the primary curve and all the secondary compensatory deviations.

We should also acknowledge that, methodologically, the discrepancy in therapist interaction time between the two groups introduces a potential performance bias, making it challenging to completely isolate the purely biomechanical effects of the Schroth exercises from the psychological benefits derived from continuous therapist support.

Treatment compliance regarding both brace wear and home exercise program was assessed through self-reported data rather than objective digital monitoring. This approach carries the risk of recall or social desirability bias, potentially leading to overestimated adherence rates [89].

Although the outcome measures assess distinct physiological domains, the testing of multiple dependent variables within the same dataset without multiplicity adjustments (such as the Bonferroni correction) inherently inflates the risk of Type I errors. This statistical limitation should be considered when interpreting the significance of the findings.

Finally, the 10-week duration of the intervention and the absence of a long-term follow-up represent a limitation. Without durability data, it remains unclear whether the observed sensorimotor adaptations are permanently retained or if they are dependent on ongoing supervised therapy. Τhe clinical implications of the present findings currently remain short-term, highlighting the need for future longitudinal studies to establish the long-term retention of these benefits.

### 4.3. Future Research

Future studies should pursue a randomized design, if possible, to confirm those findings. Male participants and variability of curve patterns would improve external validity. Long-term follow-ups extending beyond 6 months are needed to assess the durability of the observed sensorimotor adaptations, determining whether they are permanently retained or if they are transient without ongoing supervised therapy. Additionally, long-term studies should integrate objective radiographic outcomes. Combining sensorimotor data with radiological evidence will help contextualize whether functional improvements directly correlate with structural curve (Cobb angle) changes. Although the equipment used in this study is accessible for clinicians, maybe other multidimensional assessment tools, such as motion analysis, would strengthen the evidence base.

## 5. Conclusions

This study suggests that the integration of a 10-week Schroth program with bracing treatment is associated with enhanced sensorimotor control in individuals with AIS. Measurable improvements in postural stability, dynamic balance and spinal and upper-limb proprioceptive accuracy suggest that Schroth-based exercise programs can address the sensorimotor control deficits that patients with AIS exhibit. Furthermore, the intervention was associated with reduced brace-induced stress and improved quality of life scores. For physiotherapists, the findings imply that these sensorimotor control indices can serve as valuable quantifiable markers for monitoring the treatment success alongside the established radiological and clinical methods. Finally, in alignment with contemporary neuromotor paradigms, the findings of this study lay a theoretical framework providing a rationale for how Schroth may contribute to spinal stability by affecting the neural control subsystem and facilitating sensorimotor recalibration.

## Figures and Tables

**Table 1:** Participant baseline characteristics.

	Schroth Group Mean (SD)	Control Group Mean (SD)	*p*
Age (Y)	13 (1.08)	13.4 (1.45)	0.27
Height (cm)	161.9 (7.82)	161.4 (6.5)	0.79
Weight (kg)	50.6 (8.5)	53.2 (10.5)	0.28
Risser Stage	2.4 (1.22)	2.26 (1.33)	0.54
Primary Curve Cobb’s Angle (°)	30.1 (5.31)	32.16 (7.16)	0.21

SD: Standard deviation; *p* values were calculated using independent samples *t*-tests for baseline group characteristic comparison.

**Table 2:** GR-BSSQ Brace, paired *t*-test within-group comparisons.

Group	Outcome Measure	Initial Measurement	Final Measurement	Mean Difference	95% CI of the Difference Lower Upper		*p*
Schroth	Score 0–24	12.26 (5.14)	16.2 (4.58)	−3.93	−5.08	−2.77	0.000
Control	Score 0–24	14.5 (5.17)	15 (5.13)	−0.5	−1.44	0.44	0.288

**Table 3:** GR-BSSQ Brace score and stress level interpretation.

GR-BSSQ Brace Initial Evaluation	Stress Level	Group	
		Schroth	Control
	Low	4	6
	Medium	18	22
	High	8	2
**GR-BSSQ Brace Final Evaluation**			
	Low	13	7
	Medium	15	20
	High	2	3

**Table 4:** GR-BSSQ Brace, two-way mixed ANOVA.

Outcome Measure	Factor	F (1,58)	Partial Eta^2^	*p*
Score 0–24	Time Effects	36.90	0.389	0.000
	Group × Time Effects	22.13	0.276	0.000
	Group Effects	0.17	0.003	0.679

**Table 5:** Static balance test, paired *t*-test within-group comparisons.

Group	Outcome Measure	Initial Measurement	Final Measurement	Mean Difference	95% CI of the Difference Lower Upper		*p*
Schroth	Sway Velocity (mm/s)	23.75 (8.74)	20.16 (9.92)	3.58	−0.23	7.4	0.065
Control	Sway Velocity (mm/s)	20.78 (7.28)	21.02 (11.04)	−0.24	−3.97	3.48	0.893
Schroth	Ellipse Area (mm^2^)	202.71 (127.66)	118.24 (78.22)	84.46	49.28	119.64	0.000
Control	Ellipse Area (mm^2^)	167.27 (144.27)	152.47 (103.28)	14.79	−19.75	49.34	0.388

**Table 6:** Static balance test, two-way mixed ANOVA.

Outcome Measure	Factor	F (1,58)	Partial Eta^2^	*p*
Sway Velocity (mm/s)	Time Effects	1.63	0.027	0.206
	Group × Time Effects	2.15	0.036	0.148
	Group Effects	0.26	0.005	0.606
Ellipse Area (mm^2^)	Time Effects	16.95	0.226	0.000
	Group × Time Effects	8.35	0.126	0.005
	Group Effects	0.00	0.000	0.982

**Table 7:** Fukuda test, paired *t*-test within-group comparisons.

Group	Outcome Measure	Initial Measurement	Final Measurement	Mean Difference	95% CI of the Difference Lower Upper		*p*
Schroth	Distance (cm)	34.9 (15.4)	28.64 (10.82)	6.24	3.57	8.91	0.000
	Angle (°)	12.9 (7.32)	11.19 (8.53)	1.7	−2.63	6.05	0.428
Control	Distance (cm)	37.04 (22.33)	36.52 (20.9)	0.52	−2.71	3.75	0.745
	Angle (°)	17.51 (10.07)	17.23 (7.59)	0.28	−2.31	2.88	0.825

**Table 8:** Fukuda test, two-way mixed ANOVA.

Outcome Measure	Factor	F (1,58)	Partial Eta^2^	*p*
Distance (cm)	Time Effects	10.88	0.158	0.002
	Group × Time Effects	7.79	0.118	0.007
	Group Effects	1.23	0.021	0.271
Angle (°)	Time Effects	0.64	0.011	0.424
	Group × Time Effects	0.33	0.006	0.567
	Group Effects	8.79	0.132	0.004

**Table 9:** Spinal lateral flexion JPS, paired *t*-test within-group comparisons.

Group	Outcome Measure		Initial Measurement	Final Measurement	Mean Difference	95% CI of the Difference Lower Upper		*p*
Schroth	Error (°)	R	3.79 (2.28)	2.21 (0.66)	1.57	0.73	2.41	0.001
		L	4.21 (2.03)	3.51 (1.58)	0.69	0.23	1.16	0.04
		Combined	4 (1.66)	2.86 (0.92)	1.13	0.61	1.66	0.000
Control	Error (°)	R	4.96 (3.22)	4.78 (3.46)	0.18	−0.56	0.92	0.625
		L	3.74 (2.09)	3.69 (1.81)	0.05	−0.56	0.68	0.848
		Combined	4.35 (2.39)	4.23 (2.34)	0.11	−0.41	0.65	0.651

JPS = joint position sense, R = right, L = left, Combined = combined means of right and left side.

**Table 10:** Spinal lateral flexion JPS test, two-way mixed ANOVA.

Outcome Measure	Factor	F (1,58)	Partial Eta^2^	*p*
Error (°) R	Time Effects	10.18	0.149	0.002
	Group × Time Effects	6.42	0.100	0.014
	Group Effects	8.87	0.330	0.008
Error (°) L	Time Effects	3.98	0.064	0.051
	Group × Time Effects	2.84	0.047	0.097
	Group Effects	0.09	0.002	0.754
Error (°) Combined	Time Effects	11.68	0.168	0.001
	Group × Time Effects	7.65	0.117	0.008
	Group Effects	3.42	0.056	0.067

JPS = joint position sense, R = right, L = left, Combined = combined means of right and left side.

**Table 11:** Upper-limb functional proprioception test, paired *t*-test within-group comparisons.

Group	Outcome Measure		Initial Measurement	Final Measurement	Mean Difference	95% CI of the Difference Lower Upper		*p*
Schroth	Error (cm)	R	2.46 (1.35)	1.67 (0.7)	0.59	0.30	0.87	0.000
		L	2.17 (0.93)	1.66 (0.4)	0.51	0.21	0.8	0.001
		Combined	2.32 (0.99)	1.76 (0.47)	0.55	0.3	0.79	0.000
Control	Error (cm)	R	2.07 (1.13)	2.33 (1.37)	−0.25	−0.51	0.002	0.052
		L	1.52 (0.71)	1.64 (0.56)	−0.11	−0.29	0.05	0.178
		Combined	1.80 (0.86)	1.99 (0.89)	−0.18	−0.34	−0.02	0.023

R = right, L = left, Combined = combined means of right and left side. Values of this table were calculated from the mean errors of apertures 1 to 8.

**Table 12:** Upper-limb functional proprioception test, two-way mixed ANOVA.

Outcome Measure	Factor	F (1,58)	Partial Eta^2^	*p*
Error (cm) R	Time Effects	3.18	0.052	0.079
	Group × Time Effects	20.42	0.260	0.000
	Group Effects	0.01	0.000	0.903
Error (cm) L	Time Effects	5.64	0.089	0.021
	Group × Time Effects	14.32	0.198	0.000
	Group Effects	4.45	0.071	0.039
Error (cm) Combined	Time Effects	6.54	0.101	0.013
	Group × Time Effects	26.82	0.316	0.000
	Group Effects	0.52	0.009	0.471

R = right, L = left, Combined = combined means of right and left side. Values of this table were calculated from the mean errors of apertures 1 to 8.

## Data Availability

The original contributions presented in this study are included in the article. Further inquiries can be directed to the corresponding author.

## Abbreviations

The following abbreviations are used in this manuscript:

AIS	Adolescent Idiopathic Scoliosis.
ATR	Angle of Trunk Rotation
PSSE	Physiotherapeutic Scoliosis-Specific Exercise.
PNF	Proprioceptive Neuromuscular Facilitation.
IS	Idiopathic Scoliosis.
OSI	Overall Stability Index.
APSI	Anterior–Posterior Stability Index.
MLSI	Medial–Lateral Stability Index.
ANOVA	Analysis of Variance.
ICC	Intraclass Correlation Coefficient.
TW3	Tanner-Whitehouse 3.
GR-BSSQ	Greek version of the Bad Sobernheim Stress Questionnaire.
CI	Confidence Interval
SEM	Standard Error of Measurement.
SDD	Smallest Detectable Difference.
COG	Center of Gravity.
JPS	Joint Position Sense.
CAD/CAM	Computer-Aided Design and Computer-Aided Manufacturing.
SD	Standard Deviation.
SEAS	Scientific Exercises Approach to Scoliosis.
QOL	Quality of Life.

## Appendix A

**Table A1:** Right upper-limb functional proprioception test, paired *t*-test within-group comparisons for apertures 1 to 8.

Group	Outcome Measure	Aperture	Initial Measurement	Final Measurement	Mean Difference	95% CI of the Difference Lower Upper		*p*
Schroth	Error (cm)	1	1.83 (1)	1.14 (0.73)	0.68	0.31	1.04	0.001
		2	2.82 (0.92)	1.74 (0.85)	1.07	0.43	1.71	0.002
		3	3.45 (2.23)	2.57 (1.37)	0.51	0.01	1.01	0.046
		4	1.52 (1.04)	0.88 (0.44)	0.63	0.33	0.93	0.000
		5	2.84 (2.14)	2.32 (1.17)	0.51	−0.10	1.14	0.002
		6	1.96 (1.22)	1.43 (0.85)	0.53	0.14	0.91	0.008
		7	2.35 (1.61)	1.82 (0.90)	0.53	0.05	1	0.028
		8	2.91 (1.96)	2.66 (1.39)	0.24	−0.10	0.6	0.167
Control	Error (cm)	1	1.37 (0.93)	1.65 (1.87)	−0.28	−0.81	0.25	0.293
		2	2.4 (1.69)	2.15 (1.64)	−0.35	−0.63	0.56	0.904
		3	3.06 (1.78)	3.07 (1.32)	−0.1	−0.57	0.55	0.971
		4	1.30 (0.83)	1.87 (1.64)	−0.58	−1.22	0.09	0.093
		5	2.66 (1.88)	3.04 (1.55)	−0.38	−0.89	0.13	0.145
		6	1.62 (1.03)	1.98 (1.59)	−0.35	−0.8	0.09	0.115
		7	2.08 (1.5)	2.17 (1.77)	−0.85	−0.4	0.23	0.585
		8	2.39 (1.71)	2.73 (1.74)	−0.34	−0.82	0.13	0.153

**Table A2:** Right upper-limb functional proprioception test, two-way mixed ANOVA.

Outcome Measure/Aperture	Factor	F (1,58)	Partial Eta^2^	*p*
Error (cm)/1	Time Effects	1.61	0.027	0.208
	Group × Time Effects	9.22	0.137	0.004
	Group Effects	0.01	0.000	0.910
Error (cm)/2	Time Effects	5.88	0.092	0.018
	Group × Time Effects	6.71	0.104	0.012
	Group Effects	0.18	0.003	0.667
Error (cm)/3	Time Effects	1.86	0.031	0.178
	Group × Time Effects	2.01	0.034	0.161
	Group Effects	0.17	0.003	0.675
Error (cm)/4	Time Effects	0.04	0.001	0.831
	Group × Time Effects	11.41	0.164	0.001
	Group Effects	3.19	0.052	0.079
Error (cm)/5	Time Effects	0.11	0.002	0.732
	Group × Time Effects	5.08	0.081	0.028
	Group Effects	0.44	0.008	0.507
Error (cm)/6	Time Effects	0.36	0.006	0.547
	Group × Time Effects	9.48	0.141	0.003
	Group Effects	0.14	0.002	0.706
Error (cm)/7	Time Effects	2.57	0.042	0.114
	Group × Time Effects	4.93	0.078	0.030
	Group Effects	0.01	0.000	0.914
Error (cm)/8	Time Effects	0.1	0.002	0.743
	Group × Time Effects	4.1	0.066	0.047
	Group Effects	0.29	0.005	0.592

**Table A3:** Left upper-limb functional proprioception test, paired *t*-test within-group comparisons for apertures 1 to 8.

Group	Outcome Measure	Aperture	Initial	Group	Outcome Measure	95% CI of the Difference Lower Upper		*p*
Schroth	Error (cm)	1	1.69 (1.24)	1.06 (0.64)	0.62	0.2	1.04	0.005
		2	2.23 (1.16)	1.8 (0.89)	0.43	−0.1	0.87	0.055
		3	1.78 (1.14)	1.37 (0.65)	0.41	−0.7	0.9	0.094
		4	1.6 (0.88)	1.01 (0.54)	0.59	0.2	0.98	0.004
		5	2.68 (1.36)	2.3 (1.33)	0.37	−0.3	1.05	0.271
		6	1.93 (1.14)	1.51 (0.84)	0.42	0.05	0.79	0.026
		7	2.88 (1.87)	2.20 (0.8)	0.68	0.11	1.24	0.20
		8	2.55 (1.66)	2 (0.91)	0.55	0.16	0.94	0.007
Control	Error (cm)	1	1.50 (1.39)	1.43 (0.68)	0.07	−0.34	0.48	0.730
		2	1.50 (1.05)	1.68 (1.15)	−0.177	−0.52	0.16	0.301
		3	1.37 (0.70)	1.34 (0.46)	0.32	−0.17	0.23	0.749
		4	1.34 (0.9)	1.30 (0.91)	0.38	−0.32	0.39	0.827
		5	1.89 (1.22)	2.13 (0.75)	−0.23	−0.57	0.09	0.161
		6	1.26 (0.76)	1.43 (0.92)	−0.17	−0.52	0.18	0.331
		7	1.83 (1.09)	1.75 (0.97)	0.07	−0.36	0.52	0.719
		8	1.51 (1.1)	2.08 (0.81)	−0.57	−0.93	−0.2	0.003

**Table A4:** Left upper-limb functional proprioception test, two-way mixed ANOVA.

Outcome Measure/Aperture	Factor	F (1,58)	Partial Eta^2^	*p*
Error (cm)/1	Time Effects	5.84	0.092	0.019
	Group × Time Effects	3.73	0.060	0.058
	Group Effects	0.15	0.003	0.693
Error (cm)/2	Time Effects	0.87	0.015	0.355
	Group × Time Effects	4.94	0.079	0.030
	Group Effects	3.19	0.052	0.079
Error (cm)/3	Time Effects	2.95	0.049	0.041
	Group × Time Effects	2.16	0.036	0.147
	Group Effects	2.03	0.034	0.159
Error (cm)/4	Time Effects	5.96	0.093	0.018
	Group × Time Effects	4.59	0.073	0.036
	Group Effects	0.01	0.000	0.911
Error (cm)/5	Time Effects	0.13	0.002	0.713
	Group × Time Effects	2.69	0.044	0.106
	Group Effects	3.74	0.061	0.058
Error (cm)/6	Time Effects	1	0.017	0.319
	Group × Time Effects	5.64	0.089	0.021
	Group Effects	0.07	0.055	0.072
Error (cm)/7	Time Effects	4.69	0.075	0.034
	Group × Time Effects	2.95	0.048	0.091
	Group Effects	7.48	0.114	0.008
Error (cm)/8	Time Effects	0.00	0.000	0.943
	Group × Time Effects	18.50	0.242	0.000
	Group Effects	3.13	0.051	0.082
